# Chorioallantoic placentation in Galea spixii (Rodentia, Caviomorpha, Caviidae)

**DOI:** 10.1186/1477-7827-6-39

**Published:** 2008-09-04

**Authors:** Moacir F Oliveira, Andrea Mess, Carlos E Ambrósio, Carlos AG Dantas, Phelipe O Favaron, Maria A Miglino

**Affiliations:** 1 Department of Animal Science, Universidade Federal Rural do Semi-Árido, Costa e Silva 59625-900, Caixa-Postal: 137, Mossoró, Rio Grande do Norte, Brazil; 2 Museum of Natural History, Department of Research, Humboldt-University Berlin, Invalidenstr. 43, D-10115 Berlin, Germany; 3 Department of Surgery, Faculty of Veterinary Medicine, University of Sao Paulo, Av. Prof. Dr. Orlando Marques de Paiva, no. 87, Cidade Universitária, São Paulo, SP, CEP 05508-000, Brazil

## Abstract

**Background:**

Placentas of guinea pig-related rodents are appropriate animal models for human placentation because of their striking similarities to those of humans. To optimize the pool of potential models in this context, it is essential to identify the occurrence of characters in close relatives.

**Methods:**

In this study we first analyzed chorioallantoic placentation in the prea, Galea spixii, as one of the guinea pig's closest relatives. Material was collected from a breeding group at the University of Mossoró, Brazil, including 18 individuals covering an ontogenetic sequence from initial pregnancy to term. Placentas were investigated by means of histology, electron microscopy, immunohistochemistry (vimentin, α-smooth muscle actin, cytokeration) and proliferation activity (PCNA).

**Results:**

Placentation in Galea is primarily characterized by an apparent regionalization into labyrinth, trophospongium and subplacenta. It also has associated growing processes with clusters of proliferating trophoblast cells at the placental margin, internally directed projections and a second centre of proliferation in the labyrinth. Finally, the subplacenta, which is temporarily supplied in parallel by the maternal and fetal blood systems, served as the center of origin for trophoblast invasion.

**Conclusion:**

Placentation in Galea reveals major parallels to the guinea pig and other caviomorphs with respect to the regionalization of the placenta, the associated growing processes, as well as trophoblast invasion. A principal difference compared to the guinea pig occurred in the blood supply of the subplacenta. Characteristics of the invasion and expanding processes indicate that Galea may serve as an additional animal model that is much smaller than the guinea pig and where the subplacenta partly has access to both maternal and fetal blood systems.

## Background

**Figure 1:**
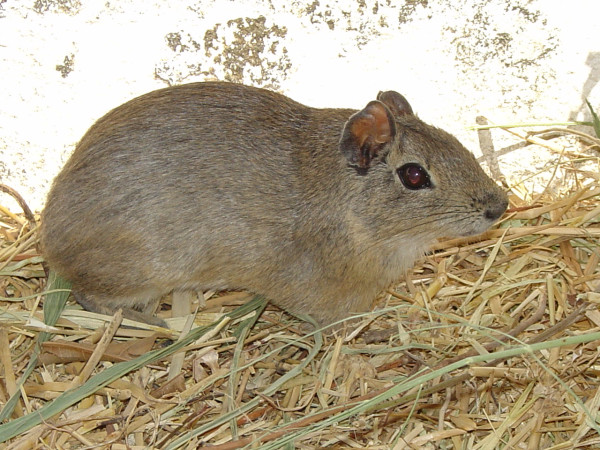
**The prea, *Galea spixii***. An adult individual at the Universidade Federal Rural do Semi-Árido, Mossoró, Brazil. As a close relative of the guinea pig, this species has a standard length of only 22.5 to 23.5 cm, an average weight of just 375 to 405 g as well as a gestation period of 48 days and litter sizes of only 2 to 4 young [21-24].

Animal models are essential to investigate aspects of human placentation [1]. Thanks to striking similarities, the guinea pig *Cavia porcellus *is regarded as an appropriate model for haemochorial placentation, the associated fetomaternal exchange and placental growth processes as well as trophoblast invasion by analogy to the cell columns in the human placenta [1-7]. To expand the pool of potential models for specific topics, several of its relatives were recently studied [7-15], supplementing previous work [16-18]. As a result, a diversity of placental characters have now been identified for guinea pig-related rodents. However, some of the closest relatives of the guinea pig, species of the genus *Galea *[19,20], have not yet been studied. Here, we investigated placental development in the prea, *Galea spixii *Wagler, 1831 (Fig. 1), analyzing stages from initial pregnancy to term by means of histology, immunohistochemistry, proliferation activity and electron microscopy. With a standard length of 22.5 to 23.5 cm and an average weight of 375 g and 405 g in males and females respectively, this species is much smaller than the guinea pig. In addition,*Galea *exhibits lower litter sizes of only 2 to 4 young and a shorter gestation period of 48 days [21-23]. This species is associated with the semidry to arid caatinga vegetation of northeast Brazil [21,24]. For reproduction, females build nests among rocks and vegetation. *Galea *is a highly social animal that breeds at different times of the year, even in the dry summer season in the absence of reasonable food abundance. Thus, in addition to understanding of placental development carried out here,*Galea *may also provide insights into pregnancy success under deprived nutritional conditions with respect to climate change.

## Methods

Material of determinate age was collected from a breeding group at the Universidade Federal Rural do Semi-Árido, Mossoró, covering 18 individual from initial pregnancy to term (Table 1). Controlled breeding was undertaken and successful copulations were regarded as day zero of pregnancy. The experimental protocol was approved by the Bioethics Committee of the School of Veterinary Medicine, University of Sao Paulo. Primary fixation was mostly done by perfusion with 2.5% glutaraldehyde via the maternal system in situ after the animals were euthanized. Tissues for histology of all relevant placental areas were transferred to 10% formalin in 0.1 M phosphate buffer for 48 h, embedded in paraplast and sectioned at 5 μm using an automatic microtome (Leica RM2155, Germany). Sections were stained with haematoxylin and eosin (HE), Masson's trichrome and the periodic acid-Schiff reaction (PAS) and were examined with an Olympus BX40 microscope (Zeiss KS400 image analysis system 3.4). Tissues for transmission electron microscopy, i.e. samples from various regions such as the labyrinth, trophospongium or subplacenta, were maintained in 2.5% glutaraldehyde for 48 h, post-fixated for 2 h in 2% phosphate-buffered osmium tetroxide (pH 7.4) and embedded in Spurr's Resin. Ultrathin sections were made on an automatic ultramicrotome (Ultracut R, Leica Microsystems, Germany), contrasted with 2% uranyl acetate and 0.5% lead citrate and studied in a transmission electron microscope (Morgagni 268D, FEI Company, The Netherlands; Mega View III camera, Soft Imaging System, Germany). Immunohistochemistry was performed for vimentin to identify mesenchymal cells and stromal decidua and for α-smooth muscle actin to identify vessel walls, following methods established as standard for guinea pig-related rodents [3,6-8,13-15,25]. Additional immunohistochemistry was carried out for cytokeratin to identify epithelial cells and trophoblast, but this method did not result in fully specific reactions for guinea pig-related rodents [3,7]. As a proliferation marker, a mouse monoclonal antibody to human anti-PCNA (proliferating cell nuclear antigen) was used. Sections were rehydrated in an ethanol series during the course of which they were submitted to endogenous peroxidase blockage in 3% hydrogen peroxide (v/v) in ethanol for 20 minutes. They were then placed in 0.1 M citrate buffer, pH 6.0, and submitted to microwave irradiation at 700 MHz for fifteen minutes. Sections were equilibrated in 0.1 M phosphate-buffered saline (PBS), pH 7.4, and non-specific binding was blocked using Dako Protein Block for 20 minutes. Tissues were incubated with primary antibodies overnight at 4°C in a humid chamber. Mouse monoclonal anti-human primary antibodies were used to detect vimentin; the cytokeratin was detected by a rabbit polyclonal antibody, α-smooth muscle actin and the PCNA were performed by mouse monoclonal anti-human primaries antibodies. The slices were then rinsed in PBS and incubated with the biotinylated secondary antibody for 45 minutes, followed by streptavidin-HRP for 45 minutes). After rinsing in PBS, the binding was visualized using aminoethyl carbazole as the chromagen. Sections were counterstained with haematoxylin and mounted in Faramount^®^. Negative controls were performed using goat anti-Mouse IgG as the primary antibody solution. For complementary data see Table 2. Readers unfamiliar with placental terminology may refer to Kaufmann and Davidoff [27] and Kaufmann [5] for background information.

**Table 1:** Investigated material of *Galea spixii*

**Phase of pregnancy**	**Number of animals**	**Gestation-al days**	**Crown-rump length (CRL)**	**Diameter of the placenta**
Initial pregnancy	3	< 9	< 10 mm	4 – 8 mm
Early pregnancy	3	9 – 12	11 – 28 mm	9 – 12 mm
Mid gestation	7	16 – 32	36 – 73 mm	13 – 18 mm
Near term to term	5	36 – 48	76 – 110 mm	20 – 35 mm

Investigated stages used by the authors in this study. All material was derived from a breeding group at the Universidade Federal Rural do Semi-Árido, Mossoró, Rio Grande do Norte, Brasil and is housed at the University of Sao Paulo, Faculty of Veterinary Medicine, Sao Paulo, Brasil.

**Table 2:** Immunohistochemistry. Complete description data related to the performed immunohistochemistry reactions

**Data Reaction**	**Company**	**Origin**
Dako Protein Block	DakoCytomation	Carpinteria, CA, U.S.A
Vimentin (Type: V9, sc-6260), Dilution: 1:200	Santa Cruz Biotechnology	Santa Cruz, California, U.S.A.
Cytokeratin (PU071-UP), Dilution 1:500	Biogenex	San Ramon, California, U.S.A.
A-smooth muscle actin (Clone 1A4), Dilution 1:300	DakoCytomation	Carpinteria, CA, U.S.A
PCNA (PC10, sc-56), Dilution 1:800	Santa Cruz Biotechnology	Santa Cruz, California, U.S.A.
Streptavidin-HRP; LSAB^®^+ System-HRP,	DakoCytomation	Carpinteria, CA, U.S.A.
Aminoethyl carbazole, AEC Substrate Kit	Zymed Laboratories	South San Francisco, CA, U.S.A.
Faramount	DakoCytomation,	Carpinteria, CA, U.S.A.
Goat anti-Mouse IgG (AP308F) Dilution 1:500	Chemicon International	Temecula, CA, USA

## Results

### Macroscopy

**Figure 2:**
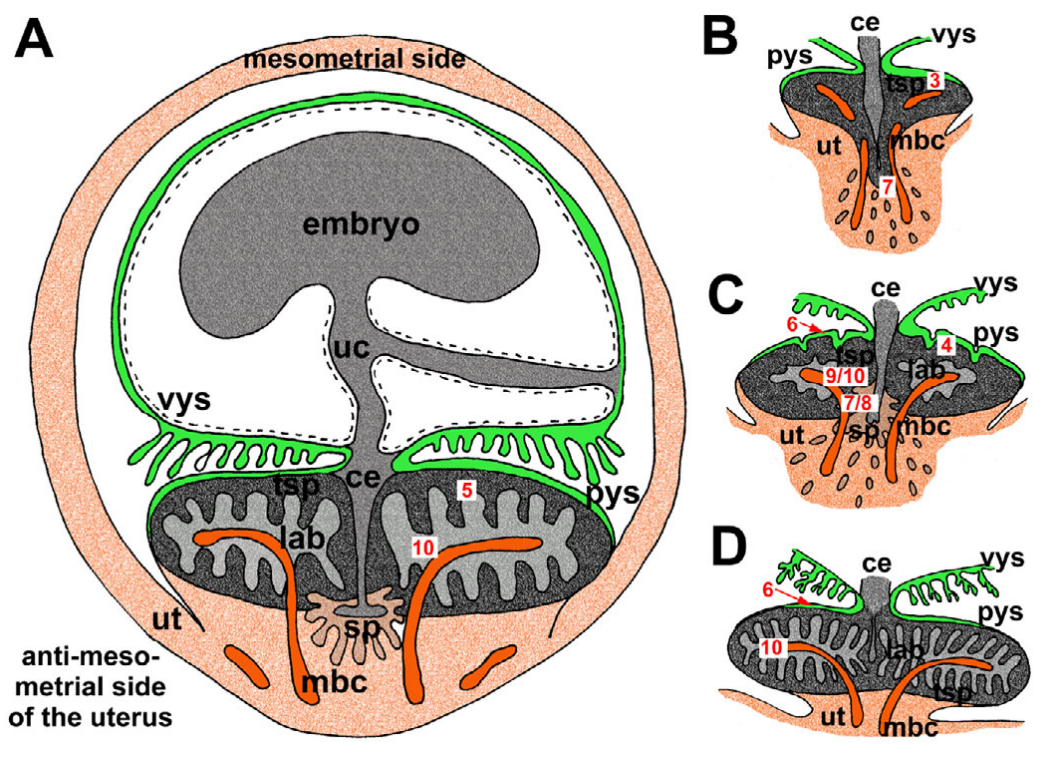
**Schematic view of the fetal membranes and the placenta**. (A) The schematic view demonstrates the arrangement of the mesometrially situated embryo, the chorioallantoic placenta, positioned at the antimesometrial side, and the other fetal membranes inside the uterus, representing an advanced stage of pregnancy. (B) In initial pregnancy, the placenta contains mainly of trophospongium with the developing subplacenta confluent to the main placenta. Strings of extraplacental trophoblast and syncytial streamers can be followed from towards the maternal blood channels. (C) In early pregnancy, a labyrinth is established and the subplacenta represents a distinct organ. (D) Near term, the placenta is highly villous with the labyrinth as the dominant area. The subplacenta has been reduced. Red numbers in white boxes refer to subsequent figures with more detailed documentation of specific regions. Ce = central excavation, lab = labyrinth, mbc = maternal blood channel, pys = parietal yolk sac, sp = subplacenta, sys = syncytial streamers, tsp = trophospongium, uc = umbilical cord, ut = uterine tissue, vys = visceral yolk sac.

**Figure 3:**
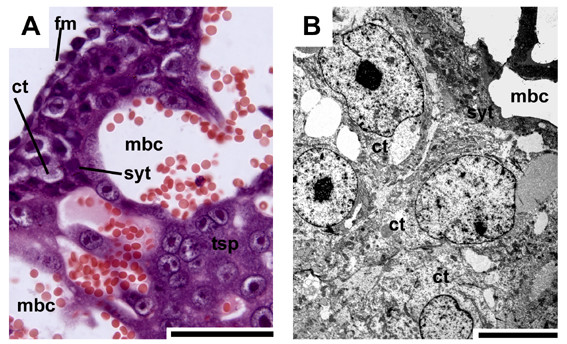
**Placental trophospongium in initial pregnancy**. (A) HE. The placenta mostly consists of trophospongium (tsp) around the maternal blood channels (mbc). Layers of cellular trophoblast (ct) are situated on fetal mesenchyme along the central excavation, covered by syncytial trophoblast (syt). (B) TEM. The trophoblast cells have large intercellular spaces in between and towards the syncytiotrophoblast. Scale bars = 0.1 mm for histology and 2 μm for TEM.

The chorioallantoic placenta is situated at the antimesometrial side of the uterus (Fig. 2A). The placenta establishes itself in initial pregnancy, i.e. before day 9 of pregnancy (Fig. 2B, see table 1). In this phase, it consists of trophospongium without fetal vessels, made by cellular and syncytial trophoblast (Figs. 2B, 3A). Strict borders to the decidua do not occur. In subsequent stages of early pregnancy, covering days 9 to 12 (see table 1), a disk with clearly defined borders to the decidua develops, which consists of trophospongium, labyrinth and subplacenta (Fig. 2C). This shape is maintained in mid gestation (represented by stages of 16 to 32 days of pregnancy, see table 1) and late pregnancy (days 36 to 48, see table 1), but in these more advanced stages the placenta is highly lobulated (Fig. 2A, D). Moreover, the labyrinth is the most prominent area and trophospongium and subplacenta are reduced in size (Fig. 2A, D).

### Growing zones

**Figure 4:**
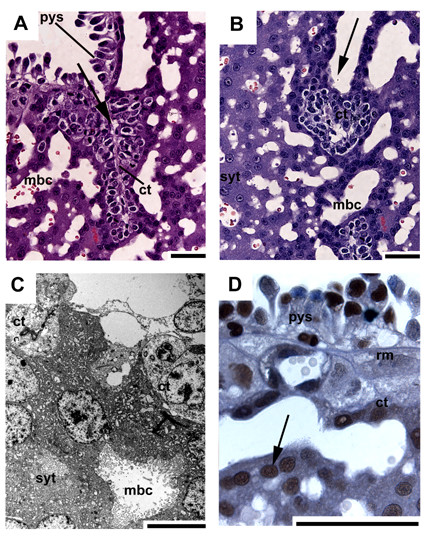
**The trophospongium in early pregnancy**. (A,B) HE. Cellular trophoblast (ct) at the placental margin (fetal side, opposite the decidua) covers internally-directed lamellae of fetal mesenchyme (arrow), situated near to the maternal blood channels (mbc). The placental surface is defined by a villous parietal yolk sac (pys). (C) TEM. Cells in the trophospongium show mitotic figures, signifying proliferation activity. (D) Immunostaining for PCNA, indicated by red nuclei, likewise reveals high proliferation activity of the trophoblast cells. Scale bars = 0.1 mm for histology and 2 μm for TEM.

**Figure 5:**
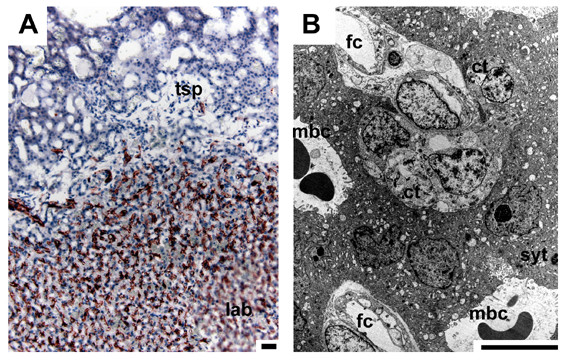
**Placental organisation in mid gestation**. (A) Immunostaining for α-actin. Only the labyrinth (lab) possesses fetal vessels (red staining of the endothelium), in contrast to the trophospongium (tsp). (B) TEM. The labyrinth includes a second centre of proliferation, represented by trophoblast cells (ct) near the fetal capillaries (fc), whereas syncytial trophoblast faces towards the maternal blood channels (mbc). Scale bars = 0.1 mm for histology and 2 μm for TEM.

In initial pregnancy layers of cellular trophoblast with noticeable borders between individual cells are situated on fetal mesenchyme as a border to the central excavation (Fig. 3A). This cytotrophoblast is followed by syncytial trophoblast, characterized by the absence of cell borders separating the cytoplasm from the nuclei, which faces towards the maternal blood channels (Fig. 3A). The trophoblast cells have large intercellular spaces in between and in relation to the syncytiotrophoblast (Fig. 3B). In early pregnancy, the trophospongium is mostly syncytial, but cytotrophoblast is still present (Fig. 4A,B). The trophoblast cells cover internally-directed lamellae of mesenchyme, reaching from the placental margin to the labyrinth (Fig. 4A,B). These cells are active in proliferation (Fig. 4C,D). The described pattern is preserved in mid gestation, but near term the amount of proliferating cells at the periphery is low. Finally, a second centre of proliferation is present in the labyrinth, which is supplied by fetal vessels (Fig. 5A), represented by cellular trophoblast within the syncytial areas (Fig. 5B).

### Periphery

**Figure 6:**
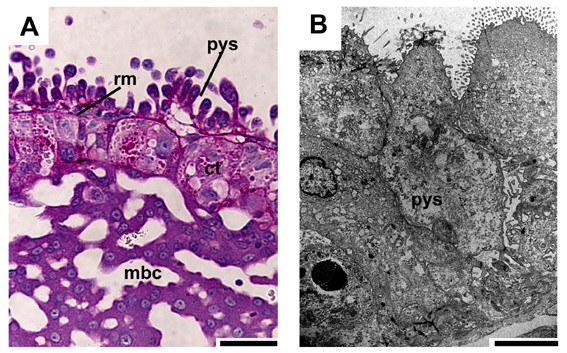
**The parietal yolk sac**. (A) Early pregnancy, PAS, counterstained with HE. The cytotrophoblast below the parietal yolk sac (pys) and the Reichert's membrane (rm) contains glycogen or related substances and may be active in secretion. Below is the trophospongium with its zone of proliferating trophoblast cells (ct) and the maternal blood channels (mbc). (B) Near term, TEM. The parietal yolk sac is still multilayered. It possesses apical microvilli, electron-dense inclusions and large intracellular spaces. Scale bars = 0.1 mm for histology and 2 μm for TEM.

The outer surface is defined by a multilayered, villous parietal yolk sac that is associated with a well-developed Reichert's membrane (Fig. 6A). Even in mid gestation and near term the parietal yolk sac appears to a large extent as a multilayered structure (Fig. 6B). The parietal yolk sac cells possess electron-dense inclusions, large intracellular spaces and apical microvilli (Fig. 6B).

### Subplacenta

**Figure 7:**
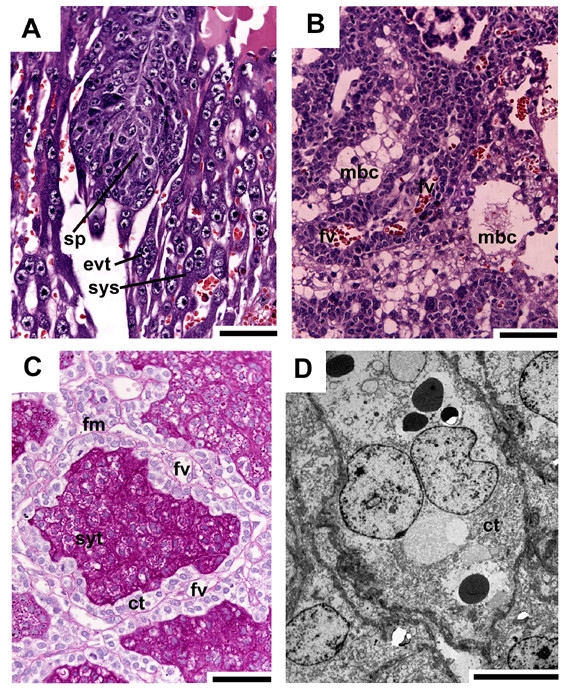
**The subplacenta**. (A) Initial pregnancy, HE. Originating from the subplacenta (sp), extraplacenta or extravillous trophoplast (evt) and syncytial streamers (sys) continue to the decidua. (B) Early pregnancy, HE. The subplacenta is supplied by fetal vessels (fv) and maternal blood channels (mbc). (C) Same stage, PAS, counterstained with HE. Each fold inside the subplacenta possesses a band of fetal mesenchyme (fm), including fetal vessels, lined with highly proliferative cytotrophoblast (ct). On the other side, syncytial trophoblast (syt) occurs that has accumulated 1–2 glycol containing substances, e.g. carbohydrates such as glycogen or mucopolysacharides, or glycoproteins or proteoglycans. (D) Same stage, TEM. Subplacental cytotrophoblast is active in proliferation. Scale bars = 0.1 mm for histology and 2 μm for TEM.

**Figure 8:**
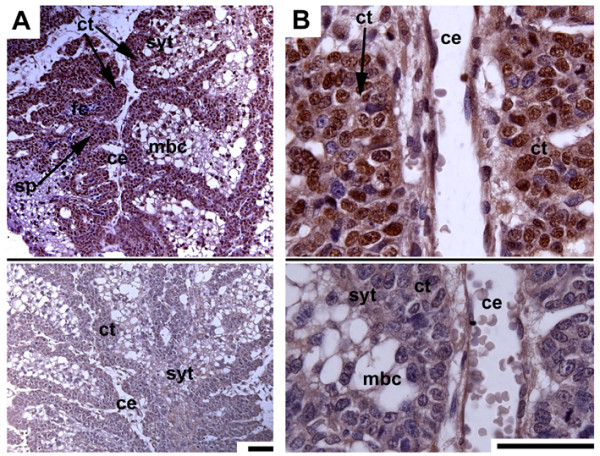
**Proliferation of subplacenta trophoblast**. (A,B) Early pregnancy, PCNA, negative IgG controls below. The subplacenta (sp), which is highly lobed and situated around the central excavation (ce), shows a high degree of proliferation activity, related to the layer of cellular trophoblast (ct). The syncytial trophoblast (syt) on the other side of the lobes faces towards the maternal blood channels (mbc). Some fetal capillaries (fc) are already present. Scale bars = 0.1 mm.

Initially the subplacenta is confluent with the main placenta and in contact with the maternal blood channels at its outer borders (Figs. 2B, 7A). Later it is a distinct organ (Fig. 2A,C). In early pregnancy and in one of the mid gestation stages, the subplacenta possesses both fetal vessels and maternal blood channels (Fig. 7B). In these cases only some, but not all of the maternal blood spaces are occluded by detritus. In all other investigated stages of mid gestation, the subplacenta is supplied by fetal vessels only. Near term, the organ is reduced and only remnants of its tissue can be found (Fig. 2D). Similar to the main placenta, the subplacenta starts as layers of cellular and syncytial trophoblast located on fetal mesenchyme. From early pregnancy on, the subplacenta is highly folded (Figs. 2A,C, 7B,C, 8A). The cytotrophoblast is highly proliferative (Figs. 7D, 8A,B). The syncytiotrophoblast contains high levels of 1–2 glycol (Fig. 7C), which indicates the presence of carbohydrates such as glycogen or mucopolysacharides as well as glycoproteins or proteoglycans. Originating from the subplacenta, strands of extraplacental trophoplast and syncytial streamers continue to the decidua (Figs. 2B,C, 7A) and the maternal blood channels. They are widespread in early pregnancy, but rare in mid gestation and absent near term (Fig. 2A,D).

### Labyrinth

**Figure 9:**
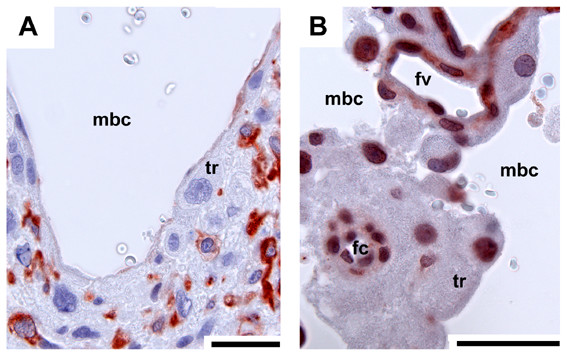
**The maternal blood channels**. Early pregnancy, immunostaining for vimentin (A) and α-actin (B) reveals the presence of trophoblast (tr) in the walls of the maternal blood channels (mbc) in the labyrinth (negative results), in contrast to fetal capillaries (fc) and larger fetal vessels (fv) that are enclosed by endothelium (red nuclei). Scale bars = 0.1 mm.

**Figure 10:**
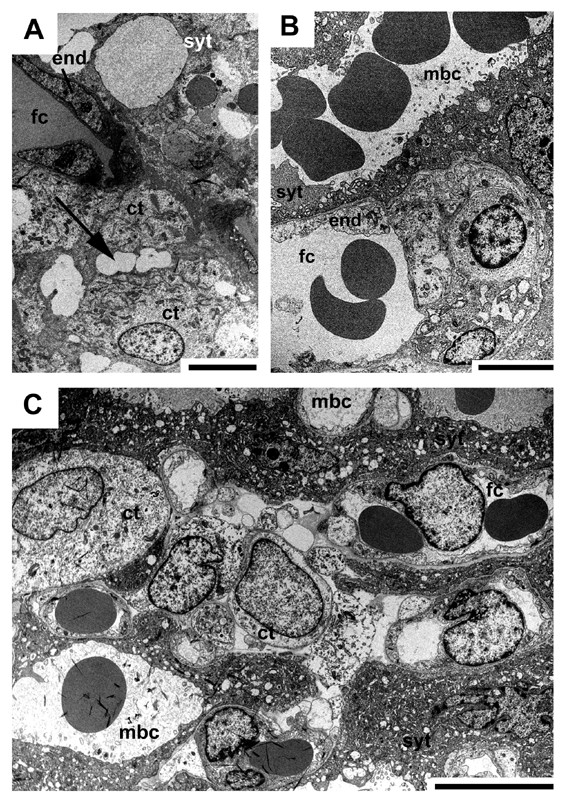
**The fetomaternal interface inside the labyrinth**. (A) Initial pregnancy, TEM. Intact endothelium (end) of a fetal capillary (fc), associated with cytotrophoblast (ct) with large intercellular spaces (arrow) and syncytial trophoblast (syt) towards the maternal blood channels (mbc). (B) Mid gestation, TEM. The interhaemal barrier is very thin. (C) Near term, TEM. Cytotrophoblast is still present in the labyrinth. Scale bars = 2 μm.

A labyrinth as an interface between the fetal and maternal blood systems arises as a distinct region in early pregnancy (Fig. 2C). At this stage, the endothelium of the maternal arteries has been destroyed and removed by trophoblast, as revealed by immunostaining for vimentin to identify mesenchymal cells and smooth α-actin to trace vessel walls (Fig. 9A,B). Positive records found by performing cytokeratin reveal this finding (but see methods and [7]). By contrast, fetal capillaries are enclosed by endothelium (Figs. 9B, 10A–C). The capillaries are associated with cellular trophoblast possessing large intercellular spaces and some syncytial trophoblast towards the maternal blood channels (Fig. 10A). From mid gestation on, syncytial trophoblast is more frequent and becomes dominant (Fig. 10B). The syncytiotroblast between the fetal and maternal blood system is, partly, very thin (Fig. 10B,C). However, even near term some cellular trophoblast is present (Fig. 10C).

## Discussion

Placental development in *Galea spixii *is, in principle, equivalent to that of the guinea pig. Similarities include a highly lobulated, labyrinthine placenta that includes labyrinth, trophospongium and subplacenta as distinct areas with a characteristic structure [5,10,11], an invasive haemochorial placental type with a thin interhaemal barrier [1-3,5,7,10], trophoblast invasion related to the subplacenta that is functionally analogous to the cell columns in the human placenta [2,5,7], trophoblast cell clusters as growing zones at the periphery and in the labyrinth [6] and the presence of the parietal yolk sac and Reichert's membrane [5]. These features are typical for placentation in caviomorph rodents and represent the ancestral condition or stem species pattern of the group [6,10,12,26,33]. However, two differences were observed. Primarily, *Cavia *possesses no coexistence between the fetal and maternal blood supply of the subplacenta [5,27-29]: supply by the maternal arterial system is stopped by coagulation, resulting in detritus-filled spaces and collapse of the lacunes, once the fetal capillaries achieve access to the subplacenta. This condition was regarded as typical for caviomorphs, although mostly only a few stages were studied [8-18]. In two individuals of *Galea *in early pregnancy and at mid gestation, both fetal and maternal blood systems are present inside the subplacenta with only some, but not all, of the maternal blood spaces occluded by detritus. The data indicate coexistence between both blood systems. Such a condition was recently documented for another caviomorph species, the degu *Octodon degus*, in which fetal vessels first arise while maternal blood lacunae are still present [11]. Moreover, very recent data indicate an overlap for the capybara too [Kanashiro et al., pers. comm.]. In all three species the maternal and fetal blood systems are separated by several layers of fetal mesenchyme, cytotrophoblast and syncytiotrophoblast, suggesting that the labyrinth is more appropriate for fetomaternal exchange [11]. Thus far, no functional or physiological significance for the overlap in the blood supply of the subplacenta is available. However, according to PAS-staining the subplacental syncytiotrophoblast accumulates glycogen or related substances such as mucopolysacharides, glycoproteins or proteoglycans. This may indicate a secretory function, e.g. for growth factors, hormones or cytokines which might be given to the fetal unit to shut down physiological functions that are not needed during fetal life. Otherwise it could have been accumulated after degeneration of the subplacenta and may be involved in the forthcoming birth, as was previously discussed by several authors [5,11,15,29-32]. Finally, even in late pregnancy the parietal yolk sac is partly multilayered in *Galea *and related species [12-18], whereas this condition has been lost in *Cavia *[5]. In this case, *Galea *exhibits the caviomorph stem species pattern, whereas *Cavia *possesses a derived character condition [26].

## Conclusion

Development of the placenta in *Galea *exhibits major parallels to the guinea pig and other caviomorphs. Due to similarities in invasion and the expanding processes, it may serve as an additional animal model for human placentation. *Galea *is smaller than the guinea pig and may thus be beneficial for some projects. Moreover, it could serve as an alternative where the center of origin for trophoblast invasion temporarily has access to both maternal and fetal blood systems during pregnancy.

## List of all abbreviations

Ce: central excavation; ct: cellular trophoblast; end: endothelium; evt: extraplacenta trophoplast; fc: fetal capillary; fm: fetal mesenchyme; fv: fetal vessel; lab: labyrinth; mbc: maternal blood channel; pys: parietal yolk sac; rm: Reichert's membrane; sp: subplacenta; sys: syncytial streamers; syt: syncytial trophoblast; tr: trophoblast; tsp: trophospongium; uc: umbilical cord; ut: uterine tissue; vys: visceral yolk sac

## Competing interests

The authors declare that they have no competing interests.

## Authors' contributions

MO established the breeding group including the determination of the stages, helped by AD. The material was processed by MO, AM and CA, and immunohistochemistry and semi thin preparation was performed by PF. AM wrote major parts of the manuscript, supported by CA and MAM.
